# Injectable citrate-modified Portland cement for use in vertebroplasty

**DOI:** 10.1002/jbm.b.33160

**Published:** 2014-04-08

**Authors:** Gareth Wynn-Jones, Richard M Shelton, Michael P Hofmann

**Affiliations:** Biomaterials Unit, School of Dentistry, University of BirminghamSt Chad's Queensway, Birmingham, B4 6NN, UK

**Keywords:** vertebroplasty, injectable bone cement, Portland cement, setting mechanism

## Abstract

The injectability of Portland cement (PC) with several citrate additives was investigated for use in clinical applications such as vertebroplasty (stabilization of a fractured vertebra with bone cement) using a syringe. A 2-wt % addition of sodium or potassium citrate with PC significantly improved cement injectability, decreased cement setting times from over 2 h to below 25 min, while increasing the compressive strength to a maximum of 125 MPa. Zeta-potential measurements indicated that the citrate anion was binding to one or more of the positively charged species causing charged repulsion between cement particles which dispersed aggregates and caused the liquefying effect of the anion. Analysis of the hydrating phases of PC indicated that the early strength producing PC phase (ettringite) developed within the first 2 h of setting following addition of the citrate anion, while this did not occur in the control cement (PC only). Within 24 h ettringite developed in PC as well as calcium–silicate–hydrate (C–S–H), the major setting phase of PC, whereas cements containing citrate did not develop this phase. The evidence suggested that in the presence of citrate the cements limited water supply appeared to be utilized for ettringite formation, producing the early strength of the citrate cements. The present study has demonstrated that it is possible to modify PC with citrate to both improve the injectability and crucially reduce the setting times of PC while improving the strength of the cement. © 2014 The Authors Journal of Biomedical Materials Research Part B: Applied Biomaterials Published by Wiley Periodicals, Inc. J Biomed Mater Res Part B: Appl Biomater, 102B: 1799–1808, 2014.

## INTRODUCTION

Calcium silicates, namely alite and belite, constitute approximately 75 wt % of the cement clinker phase of Portland cement (PC).^1^ The hydration of these phases results in the formation of calcium–silicate–hydrate (C–S–H) which possesses more than 30 polymeric forms, all of which have the general formula CaO*_x_*·SiO_2_·H_2_O*_y_*.^2^–^4^ Formation of C–S–H occurs within a few hours after the start of cement hydration and is the most important reaction for determining long-term cement strength.^3^ Tricalcium aluminate (Ca_3_Al_2_O_6_) is a by-product of cement production and in the absence of calcium sulfate dihydrate (gypsum) causes flash setting, leading to the formation of calcium aluminate hydrate (C_4_AH_13_) that causes premature stiffening of the cement paste without adding to the structural integrity.^5^ However, in the presence of gypsum the calcium aluminate hydrates to form ettringite [Ca_3_Al(OH)_6_]_2_(SO_4_)_3_·26H_2_O, which has been shown to be a set phase important in providing early cement strength.^3^,^5^–^7^

Since PC-based mineral trioxide aggregate was Food and Drug Administration approved for endodontic use in the late 1990s, there has been an increased interest in the wider use of PC as a biomaterial. It has been investigated as a drug delivery system for antibiotics used to treat bone diseases such as osteomyelitis,^8^ as a high strength hybrid material in combination with zinc^9^ and for formation of apatite on exposure to simulated body fluid when combined with zirconium.^10^ PCs are durable, possess high compressive strengths and can set in aqueous environments such as those found *in vivo*.^11^–^13^ PC would therefore be particularly suited for load-bearing applications such as vertebroplasty, which involves the subcutaneous injection of a stabilizing bone cement into fractured or even collapsed vertebrae.^14^ Polymethylmethacrylate (PMMA) is the bone cement now routinely used for vertebroplasty, PMMA has a compressive strength of 79 MPa and a setting time of 15–20 min setting time.^15^ In order to make PC a possible candidate for use as an alternative to PMMA for vertebroplasty, the cement needs to be developed to a similar strength and setting time.^16^ There are hereby two key challenges which need to be addressed before PC can be considered as a suitable bone cement for applications such as vertebroplasty. First, the cement needs to be able to be extruded through the aperture of a needle using a force suitable for manual injection. Second, the extended setting times of PC of 3–4 h at a powder-to-liquid ratio (PLR) of 3 g mL^−1^ need to be substantially reduced.^17^ It is also important to note that PC has a naturally high compressive strength but is known to be brittle with poor fracture resistance. The addition of micro fiber material such as polypropylene has been shown to improve the materials flexural strengths and fracture toughness.^18^ There are several commercially available liquefying and accelerating additives available for PC from the building industry.^1^,^19^–^21^ However, these additives were not developed for *in vivo* use and can contain biologically harmful chemicals such as formaldehyde.^22^ The citrate anion has been investigated as both a liquefier and setting accelerant for calcium phosphate based cement systems.^23^,^24^ At low concentrations citrate is also a known PC retardant but at high concentrations has demonstrated accelerant properties.^25^ The exact mechanism of the retardant/accelerant switch is not fully understood but hydroxycarboxylic anions (like citrate) have been linked by adsorption onto the aluminate phase (C_3_A) of PC leading to cement aggregate dispersion and retardation.^26^,^27^ For *in vivo* applications citrate has also demonstrated a low toxicity and even been shown to be present within human bone matrix.^28^ The aim of the present study was to develop PC as an injectable bone cement which could set in approximately 20 min and identify the mechanisms responsible for the modified PC.

## Experimental

### Materials

High-performance PC (Mastercrete, Lafarge, UK) was prepared by sieving the powder through a 500-µm steel mesh to avoid the presence of agglomerates. The cement contains four major phases including approximately 75 wt % calcium silicate, 10 wt % of both calcium aluminate and calcium aluminoferrite and 5 wt % calcium sulfate dihydrate. Trisodium citrate, tripotassium citrate, tricalcium citrate, and citric acid (Sigma, UK) were added at concentrations between 2 and 5 wt % into the powder phase. Double distilled water was added to the cement at a PLR of 5.0 g mL^−1^ and samples were hand mixed for 1 min to produce cement slurries.

### Injectability testing

Prepared PC slurry of 5 g was transferred into a 5-mL disposable syringe and extruded through an outlet diameter of 2 mm (for each variation *n* = 4). A mechanical testing machine (Instron 1185; High Wycombe Bucks, UK) was used to apply a crosshead speed of 30 mm min^−1^ and the maximum force to the syringe plunger was restricted to 100 N. The force and crosshead speed were selected to mimic the maximum force and typical rate of extrusion used during manual injection. The amount of cement remaining within the syringe was weighed and the injectability (*I*) was calculated according to the following equation:


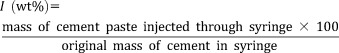
(1)

### Initial cement setting time, compressive strength, and density measurements

The initial setting times of the cements, that is the time when the cement ceases to be malleable, were measured in a normal laboratory environment (20–23°C and 50–60% humidity) using the Gilmore needle test with a needle of 113.9 g and 2.11 mm diameter according to the ASTM standard.^29^

For the compressive mechanical testing, the hand-mixed slurries were placed into polytetrafluroethylene molds producing cylindrical samples of 12 mm height and 6 mm diameter and set for 6 h. After 1 and 30 days immersion in water at 37°C the wet compressive strength of the cement samples (*n* = 30) was measured using a universal testing machine (Instron 5544; High Wycombe Bucks) at a crosshead speed of 1 mm min^−1^ (the water was not refreshed during the 30-day setting period). Dried cement strut densities were determined using helium pycnometry (10 purges, 10 runs; Accupyc 1330, Micromeritics, Bedfordshire, UK), and these data were combined with apparent wet and dry densities to calculate relative porosities.

### Investigating cement surface morphology using scanning electron microscopy

Dried cement fragments were attached onto an aluminum stub using a conducting electrodag 1415 (Agar Scientific, Stanstead UK) before being gold coated (Emitech K550X, UK). A scanning electron microscope (JEOL 7000F, Japan) was then used to image the samples at an accelerating voltage of 20 kV. Images were recorded at 2000–8000× magnification.

### Elucidating surface structures of PC using energy dispersive X-ray spectrophotometer

X-rays emitted by the elements on the surface of the cement, after irradiation with a beam of electrons were collected and recorded by a detector (Inca; Oxford Instruments, UK). CMax Software (Wycombe Bucks) was then used to analyze the elemental composition. The software assigned both a weight % (wt %) and an atomic % (at %) for each element present. From the data, the wt % ratio of each element pair was then calculated and compared with theoretical compounds known to be present during cement hydration using the following equation:


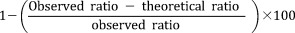
(2)

Observed wt % ratio between two elements in a cement sample. Theoretical wt % ratio between two elements in a cement sample based on empirical formulas

### Phase analysis using X-ray diffraction

X-ray diffraction (XRD) patterns of the set cements were recorded on a D8 Advance diffractometer (Bruker, Germany). Data sets were collected from 2*θ* = 5–20° with a step size of 0.02 and the count time was normalized to 1 s step^−1^. The phase compositions of the cements were determined according to the inorganic crystal structure database, calcium hydroxide (PDF Ref. 04-010-3117), calcium silicate (PDF Ref. 04-011-1393), and ettringite (PDF 00-041-1451). The calcium silicate hydrate standard was supplied by Supleco analytical (UK).

### Surface charge investigations by zeta-potential measurements

The effective surface charge of PC with various additives was measured using a Zeta-Sizer Delsa-Nano Z (Beckman-Coulter, UK). Suspensions of 0.1 g L^−1^ solid to liquid ratio of PC were hydrated just prior to the zeta-potential measurements. Sodium and potassium citrate were added at 0.2 g L^−1^ resulting in an excess of anions to bind with the PC particles. The Zeta-Sizer calculated the zeta potential by determining electrophoretic mobility using Henry's equation. For each suspension, the average zeta-potential value was obtained from five measurements.

### Infrared spectroscopy

Newly mixed PC of 1 g was placed on the diamond window of an attenuated total reflectance Fourier transform infrared spectroscopy (FTIR, Nicolet 6700; Thermo scientific) set to 23°C and covered with a glass slide with spectra recorded at 0 and 60 min. At each time point the samples were scanned 32 times with a resolution of 2.0 cm^−1^_._

### Cement enthalpy changes investigations by differential scanning calorimetry

Cement paste of 80 to 120 mg was transferred to an aluminum crucible before being inserted into the sample compartment of a differential scanning calorimeter (DSC 7; Perkin-Elmer, UK). The enthalpy change in the cement was then recorded for the next 2 h and calculated by integrating the differential scanning calorimetry (DSC) curves from the heat capacity base line using the Pyris™ DSC software (*n* = 3 for each variation).

### Statistical analysis

Data were analyzed for statistical significance using 1-way analysis of variance at a significance of p < 0.001 using SPSS statistics version 19 (IBM, UK).

## RESULTS

### Injectability studies

**FIGURE 1:**
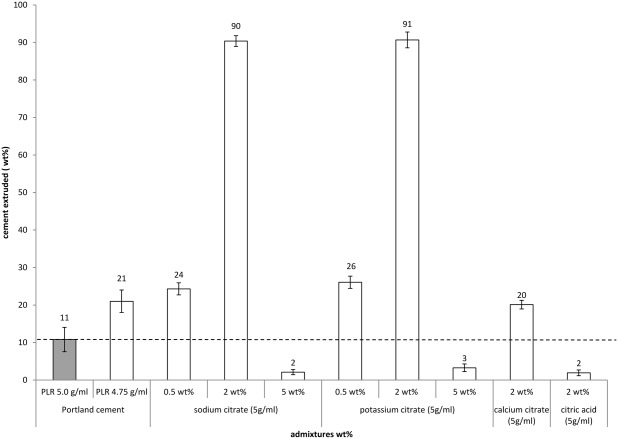
Graph showing cement extrusion in the presence of various citrate additives. Injectability significantly increased with the additions of sodium and potassium citrate up to a limit of 2 wt %. Further increasing the additions of either of these citrates to 5 wt % significantly reduced cement injectability. Calcium citrate significantly increased cement injectability compared with the PC standard, whereas citric acid significantly reduced injectability. Reducing the PLR of the cement to an equivalent of a 5-wt % addition (PLR 4.75 g mL^−1^) without an additive did not significantly increase the injectability of the cement.

Cement injectability was highest for an addition of either 2 wt % sodium or potassium citrate with both additions enabling 90 wt % cement extrusion (Figure 1). However, when increasing the additive content to 5 wt % the injectabilities significantly (p<0.05) decreased to a value of 2–3% which was even lower than the PC standard at 11%. For cement containing calcium citrate the injectability was slightly higher than the PC standard, whereas cements containing citric acid showed significantly (p<0.05) lower injectabilities. The standard cement was also injected at a PLR of 4.75 g mL^−1^ (equivalent to the PLR of PC minus a 5-wt % citrate addition) indicating that by simply increasing the water content of the cement it was not possible to significantly increase cement injectability.

**FIGURE 2:**
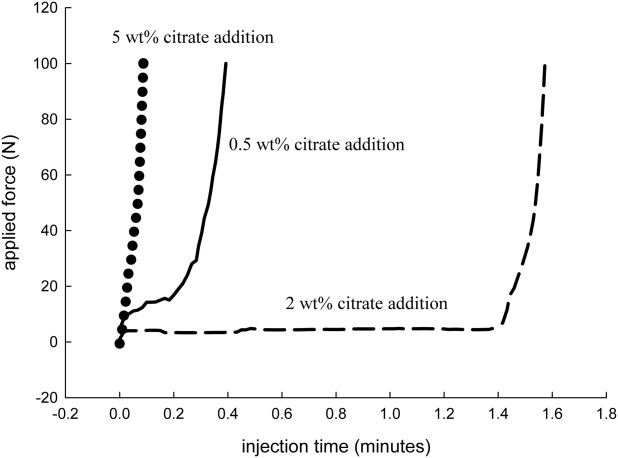
A typical force over injection time graph recorded during the injectability experiments. With a 2-wt % addition of sodium or potassium citrate a force of less than 10 N was required to extrude the cement. Altering the wt % produced reduced injectabilities.

For cements containing a 2-wt % addition of sodium or potassium citrate only a relatively low initial force of between 3 and 5 N was required to extrude the majority of the cement (Figure 2). An increase in force was required only when the syringe was nearly empty (Figure 2). In contrast, with 5 wt % sodium or potassium citrate no characteristic force plateau was evident but a sharp increase in force with little displacement as the cement failed to extrude from the syringe.

### Setting time measurements

**FIGURE 3:**
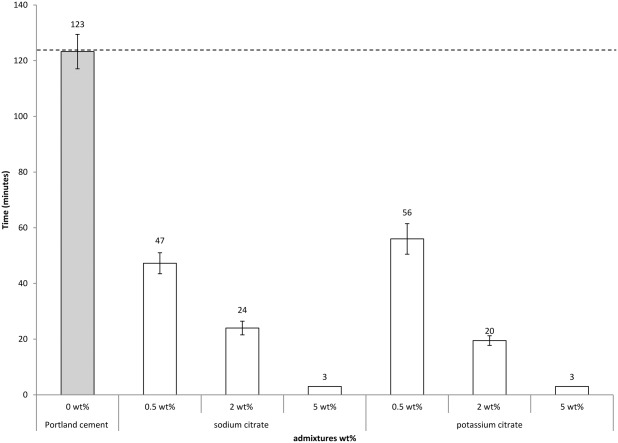
Graph showing the initial setting times of cements in the presence of 2–5 wt % sodium and potassium citrate indicating that both additives acted as accelerants.

Increasing the addition of sodium or potassium citrate from 0.5 to 5 wt % significantly reduced the initial PC setting times (from 123 min for the PC standard to 3 min with a 5-wt % addition of citrate; Figure 3). The setting time for cements containing either calcium citrate or citric acid could not be measured as the paste was too dry to assess the imprint of the Gilmore needle.

### Compressive strength, porosity, and density measurements

The highest compressive strengths were obtained by 2 wt % additions of either sodium or potassium citrate to the cement (98 and 125 MPa, respectively, after 30 days setting; Table 1). Observation indicated that calcium citrate and citric acid did not set within the molds after 6 h so compressive strength values were not measured for either of these additives.

**TABLE 1:** Compressive Strength, Relative Porosities, and Specific Densities for Cements Containing Additions of 0.5–5 wt % Sodium and Potassium Citrate

Admixture	Wt %	Compressive Strength (1 Day; MPa)	Compressive Strength (30 Days; MPa)	Relative Porosities (1 Day; %)	Relative Porosities (30 Days; %)	Specific Densities (1 Day; g cm^−3^)	Specific Densities 30 Days; g cm^−3^)
PC standard		66 ± 8	83 ± 7	19 ± 1	13 ± 1	2.62 ± 0.01	2.51 ± 0.01
Sodium citrate	0.5	57 ± 9	71 ± 7	18 ± 1	15 ± 1	2.57 ± 0.01	2.44 ± 0.01
	2	75 ± 8	98 ± 11	14 ± 1	10 ± 1	2.55 ± 0.01	2.40 ± 0.01
	5	44 ± 6	75 ± 8	19 ± 1	14 ± 1	2.54 ± 0.01	2.43 ± 0.01
Potassium citrate	2	72 ± 7	125 ± 14	13 ± 1	8 ± 1	2.54 ± 0.01	2.35 ± 0.01
	5	44 ± 7	79 ± 6	21 ± 1	13 ± 1	2.60 ± 0.01	2.39 ± 0.01

Maximum compressive strengths were achieved by the additions of either 2 wt % sodium or potassium citrate. The 2 wt % cements also possessed lower porosities and strut densities than the 0.5 and 5 wt % additions and were also lower than the PC standard.

The lowest porosities and strut densities were also observed with the 2 wt % additions of potassium and sodium citrate. For each individual additive addition the 30 day relative porosity and specific density values were found to be lower than after 1 day.

### Zeta-potential measurements

**FIGURE 4:**
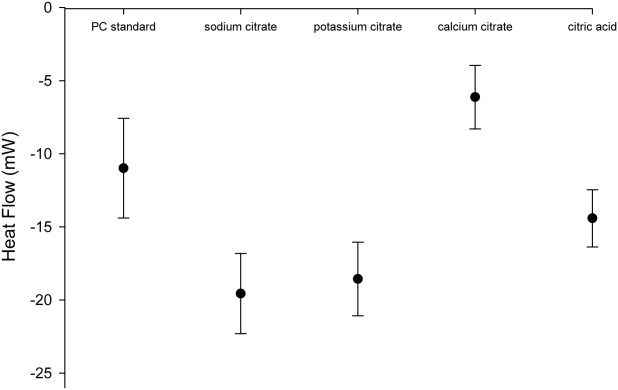
Graph showing zeta-potential measurements of PC in the presence of various citrates. Cements containing sodium or potassium citrate (−18 and −19 mV, respectively) were both significantly more negative than the PC standard (−11 mV). Citric acid (−14 mV) was also more negative than PC standard, whereas calcium citrate (−6 mV) was less negative.

The addition of sodium or potassium citrate and citric acid to PC all increased the magnitude of the negative surface charge compared with the PC standard (Figure 4). In contrast, the addition of calcium citrate reduced the magnitude of the overall negative surface charge.

### Cement enthalpy investigations by DSC

**FIGURE 5:**
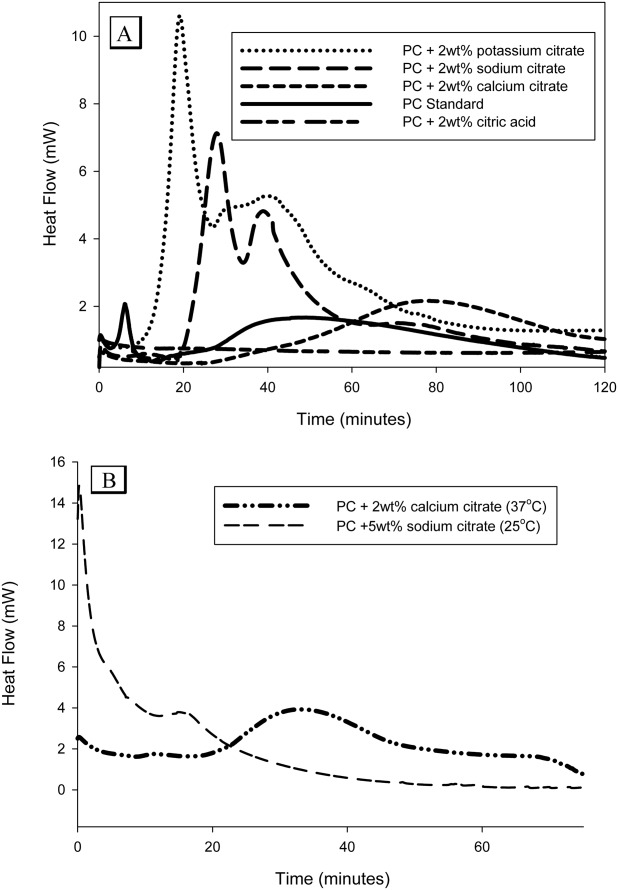
(A) Graph showing the enthalpy change in PC with 2 wt % of various citrate compounds during the first 2 h of cement hydration. The greatest enthalpy changes were observed with potassium and sodium citrate which also possessed similar profiles. (B) Enthalpy change with PC containing 5 wt % sodium citrate and calcium citrate set at 37°C. For the 5 wt % sodium citrate cement the kinetics of reaction were so fast that it was not possible to measure the initial exotherm.

The mean enthalpy changes during the hydration of PC in the presence of either 2wt % sodium or potassium citrate were 83 and 96 J g^−1^, respectively [Figure 5(a)]. Both of these values were significantly (p<0.05) higher than those obtained from the PC standard with a value of 30 J g^−1^. The heat trace signatures of potassium and sodium citrate additions to PC were also similar with an initial exotherm after approximately 20 min followed by a lower peak after approximately 40 min. For both sodium and potassium citrate additions the first peak corresponded with the initial setting of the cement which was 24 and 20 min, respectively. The addition of calcium citrate produced one exotherm with a peak after 84 min with a mean enthalpy change of 40 J g^−1^ while PC with 2 wt % citric acid did not yield any heat from the setting reaction.

An addition of 2 wt % calcium citrate to PC appeared to generate a similar but extended trace profile as seen for the sodium and potassium citrate additives [Figure 5(a)]. When the kinetics of the setting reaction of the 2 wt % calcium citrate were increased by testing the enthalpy change at 37°C a similar double peak profile was obtained [Figure 5(b)]. On addition of 5 wt % sodium citrate there was a significant acceleration of the hydration reaction which prevented measurement of the initial exotherm after sample preparation with this additive. However, based on the height and width of the initial peak the overall hydration enthalpy appeared greater than when only 2 wt % of the additive was used. The trace contained a second exotherm shoulder at 18 min which was also seen in the 2 wt % additions. The initial setting times of the 5 wt % sodium citrate cements also corresponded with the position of the first major exotherm occurring within the first 3 min of setting.

### Phase analysis using X-ray diffraction

**FIGURE 6:**
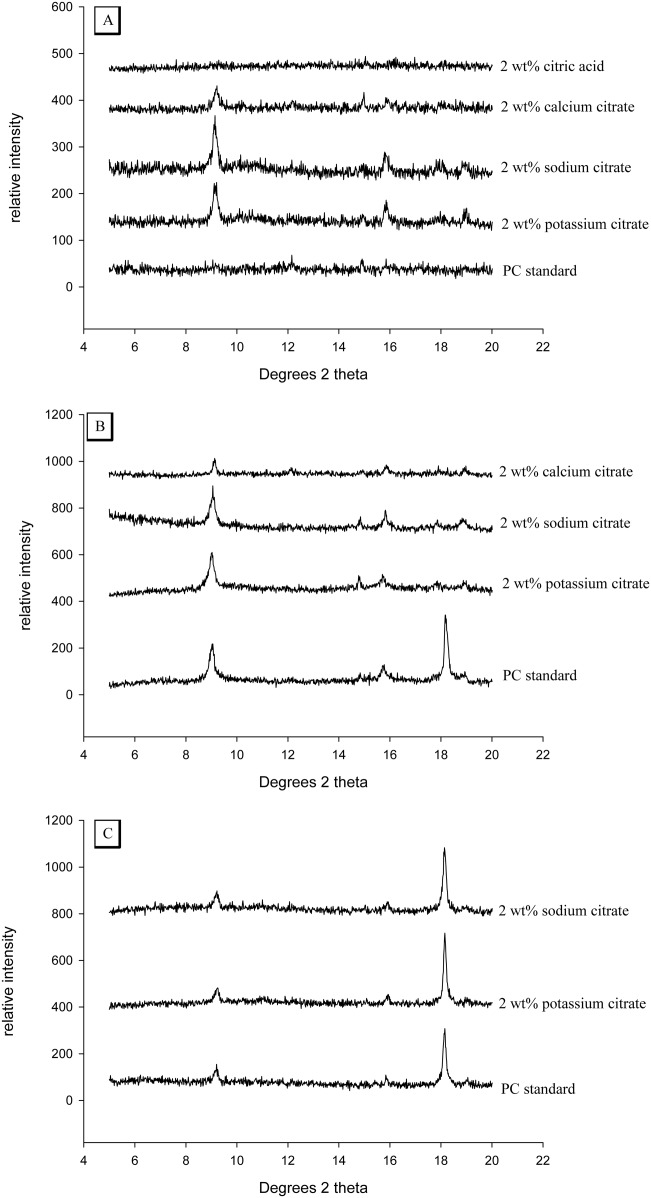
Spectra showing the X-ray diffraction patterns for PC containing 2 wt % of various citrate compounds. (A) after 2 h of setting only sodium and potassium citrates possessed ettringite peaks at 2° and 16° *θ*. (B) In contrast, after 24 h PC standard had also developed this peak in addition to another peak at 18 *θ* corresponding with Ca(OH)_2_. (C) After 30 day both standard and citrate cements possessed ettringite and Ca(OH)_2_.

There was no ettringite present in the PC standard or cements containing 2 wt % citric acid after 2 h setting as indicated by the lack of peaks at 9 and 16° *θ* (Figure 6). In contrast, cements containing 2 wt % sodium, potassium and calcium citrate possessed peaks corresponding with ettringite. After 24 h of setting the PC standard had developed an ettringite peak in addition to a calcium hydroxide peak at 18° *θ*. None of the citrate containing cements developed a calcium hydroxide peak before 24 h. Between 1 and 30 days, both the cements containing 2 wt % sodium and potassium citrate developed peaks at 18° *θ*.

### FTIR analysis

**FIGURE 7:**
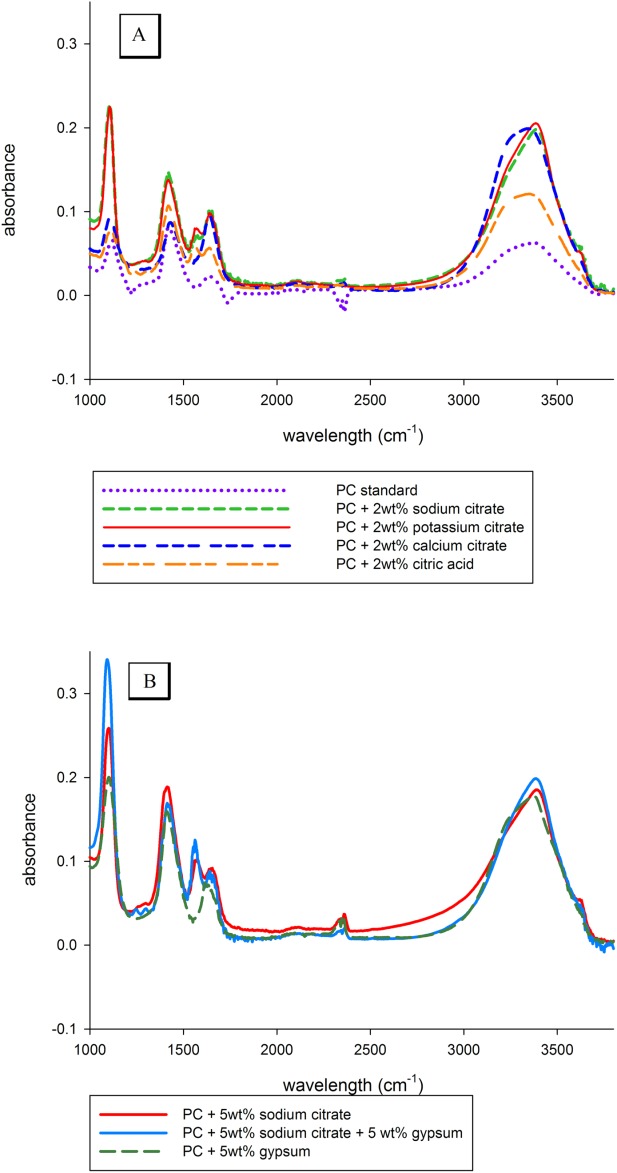
(A) FTIR spectra for PC and PC containing various 2 wt % citrates after the first hour of setting. The absorbance of the sulfate band (1100 cm^−1^) in the cements containing sodium and potassium citrate was noticeably higher than the PC standard. In contrast, the wide water peak at (3500 cm^−1^) of PC had decreased noticeably compared with the citrate containing cements. (B) When 5 wt % gypsum was added to the cement the sulfate band increased compared with the PC standard. The highest peak heights were achieved by adding gypsum in conjunction with citrate. [Color figure can be viewed in the online issue, which is available at http://wileyonlinelibrary.com.]

There was a sharp increase in the calcium sulfate dihydrate (gypsum) peak at 1100 cm^−1^ during the first 60 min of setting for cements containing 2 wt % sodium and potassium citrate [Figure 7(a)]. The *υ*_3_ absorption band of sulfate (SO_4_^2−^) is in this region and based on the profiles of standard calcium sulfates the peak related most closely to gypsum.^30^–^32^ In contrast, the gypsum peak for the PC standard and cements containing 2 wt % sodium citrate and citric acid did not increase during the first 2 h of reaction. When 5 wt % sodium citrate was added to the cement the gypsum peak was noticeably higher than with a 2-wt % addition [Figure 7(d)]. When 5 wt % gypsum was added in addition to the 5 wt % sodium citrate the gypsum peak was higher compared with when the citrate was used independently.

### Surface phase morphology using scanning electron microscopy

**FIGURE 8:**
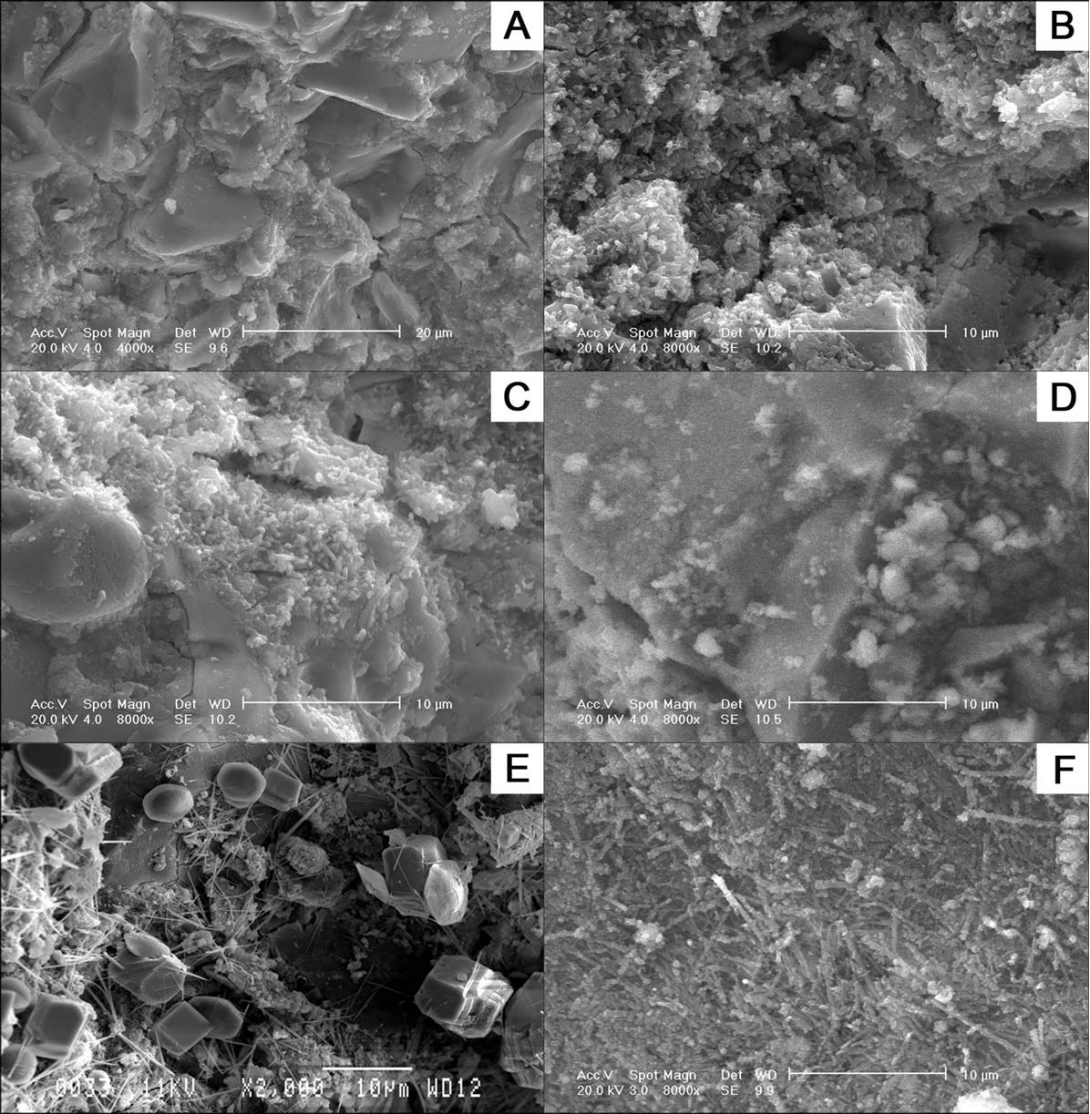
Scanning electron photomicrographs showing the surface morphology of (A) PC standard after 2 h of cement setting (B) PC containing 5 wt % sodium citrate and (C) 2 wt % potassium citrate. The addition of both of these additives produced small needle shaped crystals with 3 µm in length. (D) The addition of calcium citrate after 2 h did not produce any of these crystals. After 24 h of cement setting larger, 10 µm, needle shaped crystals were present in both the PC standard and 2 wt % sodium citrate.

Crystals approximately 2–4 µm in diameter matching the morphology of ettringite were present after 24 h setting on the cement surface containing both 2 wt % sodium and potassium citrate [Figure 8(b,c), respectively]. PC standard and cement containing 2 wt % calcium citrate did not possess any crystals of similar morphology [Figure 8(a,d), respectively]. After 24 h, the crystals on the surface of both the PC standard and the cement containing 2 wt % sodium citrate had extended to approximately 10 µm, see Figure 8(e,f), respectively.

### Elemental analysis using energy dispersive X-ray spectroscopy

When the elemental composition of the 10 µm crystals were analyzed using energy dispersive X-ray spectroscopy (EDX) there was only a 4 and 12% difference to the theoretical values for ettringite in the oxygen/aluminum and silicon/oxygen ratios, respectively.

## DISCUSSION

### Injectability studies

The overall negative surface charge of PC has been previously attributed to the ionization of the silanol groups of C–S–H (the major phase of PC) [Eq. (3)] as a consequence of PC and C–S–H sharing similar zeta potentials (C–S–H ranges from −12.7 to −9.4 mV and hydrated PC has a value of approximately −10 mV).^33^–^36^


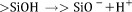
(3)

The −11 mV surface charge of the hydrating PC obtained during the present study was also similar to the known range of C–S–H surface charge indicating that the hydrating surface of PC may also be dictated by the calcium silicate phase. The increase in the magnitude of surface charge of PC in the presence of sodium and potassium citrate and citric acid (−19, −18, and −14 mV, respectively) indicated the adsorption of the citrate anion onto the surface of hydrating PC. Once bound, the resulting electrostatic repulsion force between similarly negatively charged cement particles may have caused cement aggregate dispersion thus leading to improved injectability.^37^–^39^ The reduced charge magnitude of cements containing calcium citrate may have been due to binding of the calcium ion to PC which is known to decrease the overall surface charge of hydrated PC [Eq. (4)].^34^,^35^


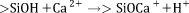
(4)

### Early acceleration of the PC setting reaction in the presence of sodium citrate

At low concentrations sodium citrate has been previously described to be a cement retardant but surprisingly acted as an accelerant when used at high concentrations, however, the underlying mechanism was never explained.^25^ The cement surface morphology (scanning electron microscopy), surface structure composition (EDX) and phase analysis (XRD) studies of PC, all independently indicated that the early accelerating effect of citrate may be caused by the formation of ettringite, [Ca_3_Al(OH)_6_]_2_(SO_4_)_3_·26H_2_O. Ettringite has been previously shown to be essential for generating the early strength of PC.^5^ Ettringite was identified in the citrate modified cements after 2 h but not in standard cements, with both cements containing this phase after 24 h. The ettringite crystals in the presence of citrate did not develop the typical “spherulite” structures produced by artificially seeding low densities of pure ettringite crystals.^40^ However, it has been previously demonstrated that the size and density of the needle shaped crystals of pure ettringite can be influenced by additives.^40^,^41^ In particular, one previous study investigating the effect of sodium citrate on pure ettringite demonstrated that citrate produced “small, short needle,” shaped ettringite crystals due to increased crystal nucleation.^41^ The central “spherulite” core may simply have been masked by crystal density (see Figure 8). The initial lack of any exotherm generated during the first 15–20 min with any 2 wt % citrate additive indicated that citrate binding and consequent aggregate dispersion prevented early bond formation leading to a retardation effect (see Figure 5).

In addition to ettringite formation the very short setting time of cement with a 5-wt % addition of citrate also indicated that the additive may have been causing the cement to flash set. Cements typically contain approximately 5 wt % calcium sulfate dihydrate (gypsum) to coat the calcium aluminate phase thus preventing early hydration (flash setting) which leads to a weak cement structure.^3^ The increase in absorbance of the FTIR unbound gypsum peak at approximately 1100 cm^−1^ occurring during the first 60 min of cement setting when 5 wt % sodium citrate was added to PC indicated that citrate at this concentration was removing gypsum from the surface of the aluminate particles altering the quantity of bound/unbound gypsum [see Figure 7(b)].^42^ Removing gypsum from the calcium aluminate phase then resulted in the formation of calcium aluminate hydrate (C_4_AH_13_), the cause of flash setting. This hypothesis was tested by the addition of gypsum to the cements containing 5 wt % sodium citrate where the rapid onset of cement setting was prevented by providing an additional source of gypsum to coat the aluminate phase. The increased height of the absorbance peak after 60 min when gypsum and sodium citrate were combined in equal wt % indicated that at 5 wt % the citrate anion had the capacity to remove more gypsum from the aluminate phase than was contained within the cement. The decreased height of the gypsum peak after 60 min of setting when only 2 instead of 5 wt % addition of sodium citrate was used indicated a decrease in the removal of gypsum from the aluminate phase. In small quantities, citrate appeared to accelerate the reaction of tricalcium aluminate with gypsum to form ettringite but in the case of high concentrations of citrate all the gypsum was removed from the aluminate causing flash setting.

Sodium citrate additions at both 2 and 5wt % generated an initial exothermic peak which corresponded with the initial setting times of the respective cements (see Figures 3 and 5). It is known that flash setting is an exothermic reaction and in both instances may have been responsible at least in part for early cement setting when sodium citrate was present.^3^ However, the formation of ettringite is known to be exothermic and it may be responsible for the second characteristic exotherm in the DSC measurements.^5^,^43^

The lack of a calcium hydroxide peak as a by-product of calcium silicate hydration, in the phase analysis of any cement containing citrate (Figure 6) indicated that for at least the first 24 h of setting citrate was additionally blocking C–S–H production, thus preventing the consumption of water in this reaction. The remaining water was subsequently available for increased ettringite generation that led to faster setting and high early strength of the cement.

### Long-term cement strength

The lower porosities of the cements modified with 2 wt % citrate when compared with the PC standard indicated that more water was consumed in the hydration reaction when citrate was present thus causing lower porosity (as less excess water was present in the structure to cause porosity).^44^ The low specific densities of the citrate modified cements also indicated a higher degree of hydration when compared with the PC as the more dense powder phase was hydrated into less dense material by chemically binding water, which is the cement reactant with the lowest density.

A relationship between compressive strength and relative porosity has been previously established.^45^–^47^


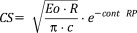
(5)where CS is the compressive strength; *E*_o_ is the modulus of zero porosity material; *R* is the fracture surface energy; *c* is the critical flaw size; and RP is the relative porosity.

If the materials constants (under the square root) remain unchanged, Eq. (5) simplifies to a linear relationship between the natural logarithm of compressive strength and relative porosity


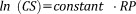
(6)

**FIGURE 9:**
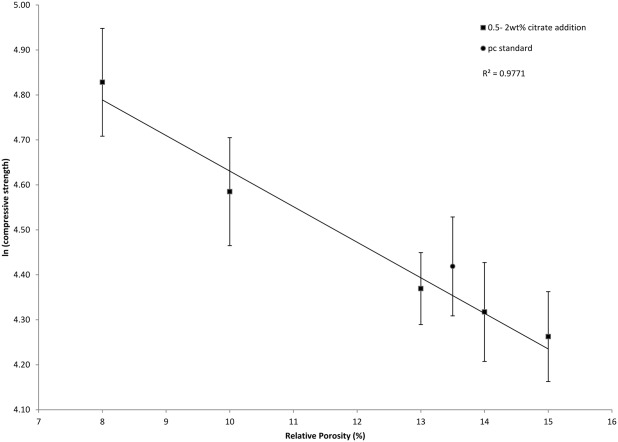
Graph showing natural log of compressive strength versus relative porosity after 30 days for 0.5–2 wt % sodium and potassium citrate. The linear relationship between the samples indicated that the additives did not alter the cement's material constants.

The natural logarithms of the compressive strength values from Table 1 were plotted as a function of relative porosity after 30-day setting. The strong linear relationship (*R*^2^ =0.97) between ln(citrate CS) and porosity (Figure 9) indicated that the material constants were not altered when the cements were modified with sodium or potassium citrate.

In summary the evidence suggested that a 2-wt % addition of sodium citrate blocked calcium silicate hydration. In turn, this left a limited amount of water in the high PLR cement (cements are usually mixed at 3 g mL^−1^ whereas 5 g mL^−1^ was used in this study), to partake in an alternative reaction such as the hydration of the calcium aluminate phase. As sodium citrate has the ability to remove gypsum from the aluminate phase, a portion of the aluminate was simply hydrated causing flash setting. However, it appeared that the major proportion of the water was utilized to produce ettringite within the first 2 h of cement setting. During the course of the following 30 days the phase analysis of the cements indicated that cements containing 2 wt % sodium or potassium citrate developed a C–S–H phase later. A combination of accelerated early ettringite formation preceding C–S–H production provides a possible explanation for the low porosities of the citrate modified cements resulting in high compressive strengths (up to 50% compared with standard PC).

### Accelerating the setting reaction of PC with other salts of citrate and citric acid

The concentration of citrate anions contained within the liquid phase of the setting cement may determine the cements setting characteristics. Sodium citrate possesses a *K*_d_ value of 0.2 *M* and potassium citrate has a marginally higher value of 0.37 *M*.^48^ Both of these citrate salts had similar influences on setting characteristics including injectability, setting times and compressive strengths (see Figures 1, 5, and 3, respectively). In contrast, calcium citrate had a lower dissociation constant of 1 m*M*. The cement possessed extended setting times and reduced injectabilities compared with the sodium and potassium salts. The reduced exotherm of calcium citrate may be another indicator of a reduced citrate concentration. When the kinetics of the setting reaction were accelerated by increasing the setting temperature to 37°C the exothermic trace had a similar profile to those of the sodium and potassium salts [see Figure 5(b)]. The phase analysis of the 2 wt % calcium citrate cements suggested that ettringite was present within the first 2 h of mixing but at a reduced quantity compared with sodium or potassium citrate. Introducing hydrogen cations from citric acid into the liquid phase may have disrupted the alkaline setting conditions of PC.^3^ The cements were observed to set as a very dry powder which produced no exotherm and also possessed strut densities similar to unhydrated PC powder.

## CONCLUSIONS

Citrate additions enabled PC injectabilities of over 90% and improved compressive strengths of up to 125 MPa compared with the PC control while reducing cement setting times to 20 min making this cement a possible alternative to PMMA. Citrate accelerated cement setting through a combination of early ettringite formation and calcium aluminate hydrate formation. This present study has demonstrated both the mechanism of citrates action with PC as well as the possibility to generate a durable high strength injectable PC based cement.
